# A Case of Multiple Plexiform Schwannomas

**DOI:** 10.7759/cureus.28140

**Published:** 2022-08-18

**Authors:** Somdatta Bhaumik, Thanka Johnson, Vinutha Gali

**Affiliations:** 1 Pathology, Sree Balaji Medical College and Hospital, Chennai, IND

**Keywords:** schwann cell neoplasm, hematoxylin and eosin, histopathology, neurofibromatosis type 2, plexiform schwannoma

## Abstract

Schwannoma is a slow-growing nerve sheath tumour comprising differentiated neoplastic Schwann cells. The plexiform variant of schwannoma grows in a plexiform or multinodular pattern and may be conventional or of cellular type. Clinically, they manifest as single, skin-coloured tumours along the distribution of peripheral or cranial nerves. This tumour usually ranges between 2 and 4 cm in size, common sites of localization being the head or the flexor aspect of the extremities. The tumour may be associated with neurofibromatosis Type 2 (but not with neurofibromatosis Type 1). The majority are biphasic tumours containing compact areas (Antoni A tissue) showing occasional nuclear palisading (Verocay bodies), alternating with loosely arranged foci (Antoni B tissue). Here, we report a case of a 37-year-old female patient, with complaints of swellings over the right forearm, right wrist and right hand. Biopsy specimens were sent from each of the swellings for histopathological evaluation. The final impression of plexiform schwannoma was made based on the microscopical examination of hematoxylin-and-eosin-stained sections.

## Introduction

Schwannomas are benign, slow-growing nerve sheath tumours composed of well-differentiated Schwann cells [1]. They are a well-circumscribed, encapsulated and loosely attached to the associated nerve without invading it. Conventional forms of schwannomas are associated with loss of expression of the neurofibromatosis Type 2 (NF2) gene product, known as merlin [1]. Plexiform schwannoma is a rare variant, which displays a plexiform or a multinodular growth pattern. It may be either conventional or cellular type. These tumors may involve multiple nerve fascicles or a nerve plexus [1]. They occur mostly in the subcutaneous tissue and are rarely confined to the dermis [2]. Clinically they manifest as a solitary, skin-coloured tumour along the course of peripheral or cranial nerves, about 2 to 4 cm in size. Usual sites include the head or the flexor aspect of the extremities [2]. Plexiform schwannomas have been diagnosed both in the childhood and at birth. Most cases are sporadic and may be associated with neurofibromatosis Type 2 [1].

## Case presentation

A 37-year-old female patient came to the surgery out-patient department with an insidious development of swellings over the right wrist joint, right hand and right forearm, which gradually progressed in size over the duration of 10 years. Clinical examination findings were a firm, 3 x 3 cm swelling over the volar aspect of the wrist joint; another firm, mobile, 4 x 3 cm swelling over the hypothenar aspect of the right hand and a third 0.5 x 0.5 cm swelling over the flexor aspect of the right forearm. Clinical diagnosis of venous malformation was suspected and a biopsy specimen from each of the swellings was sent for histopathological examination.

**Figure 1:**
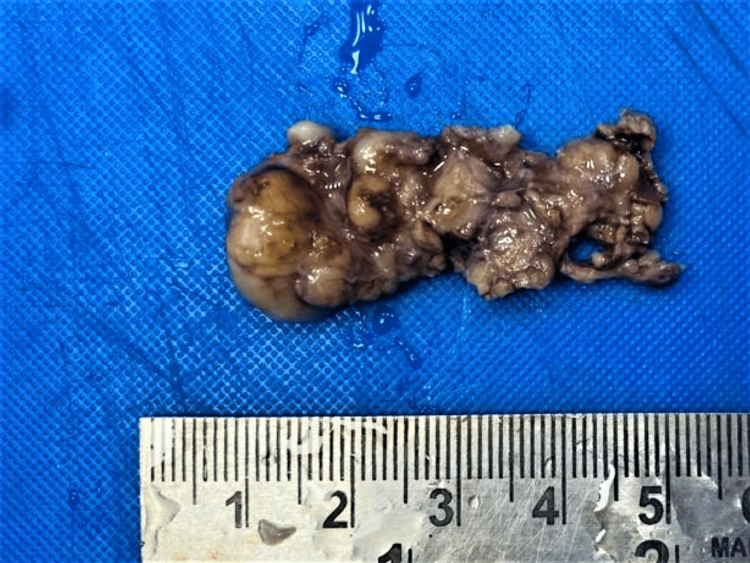
External surface of the specimen, which was grey-brown in color with a soft-to-firm consistency

**Figure 2:**
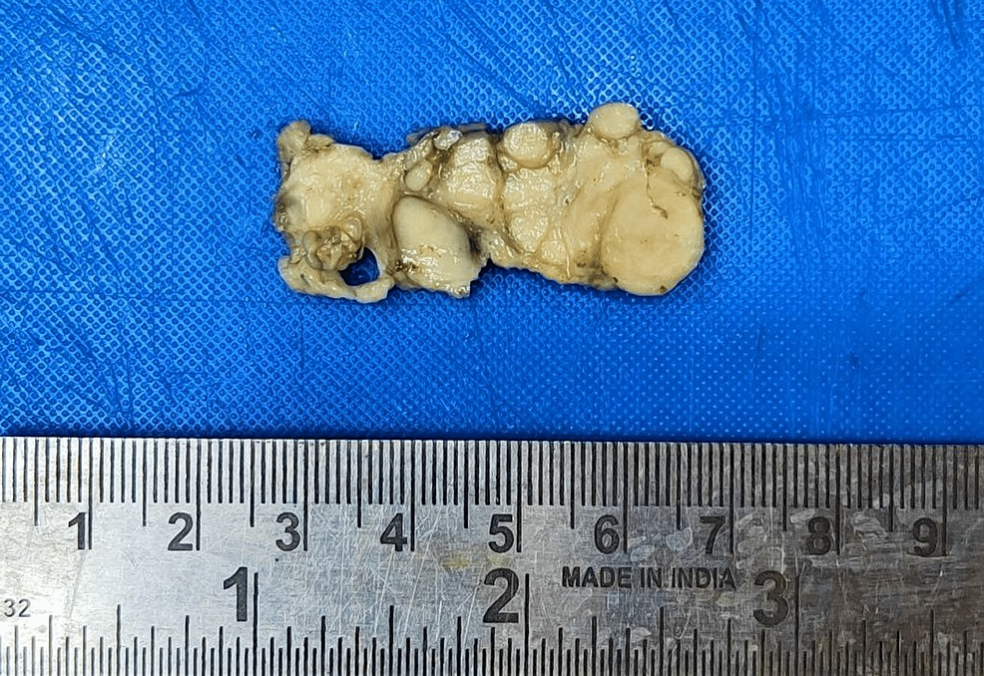
The cut-surface showing multiple grey-white nodules of varying sizes

For gross examination, three specimens (A, B and C) were received. Specimen A (swelling from the flexor aspect of the right forearm) was a single, skin-covered, grey-brown soft tissue part measuring 1 x 0.5 x 0.2 cm. The cut-surface showed a grey-white nodule 0.2 cm in diameter. Specimen B (swelling from the volar aspect of the right wrist) comprised two grey-white, soft-to-firm tissue parts, one measuring 2.6 x 0.8 x 0.8 cm and the other 2 x 0.5 x 0.5 cm. The cut-surface was homogeneously grey-white. Specimen C (swelling from the hypothenar aspect of the right hand) was a grey-brown, soft-to-firm tissue part measuring 5 x 1.5 x 1 cm (Figure 1). The cut-surface revealed multiple grey-white nodules, ranging from 0.2 to 1 cm in diameter (Figure 2).

**Figure 3:**
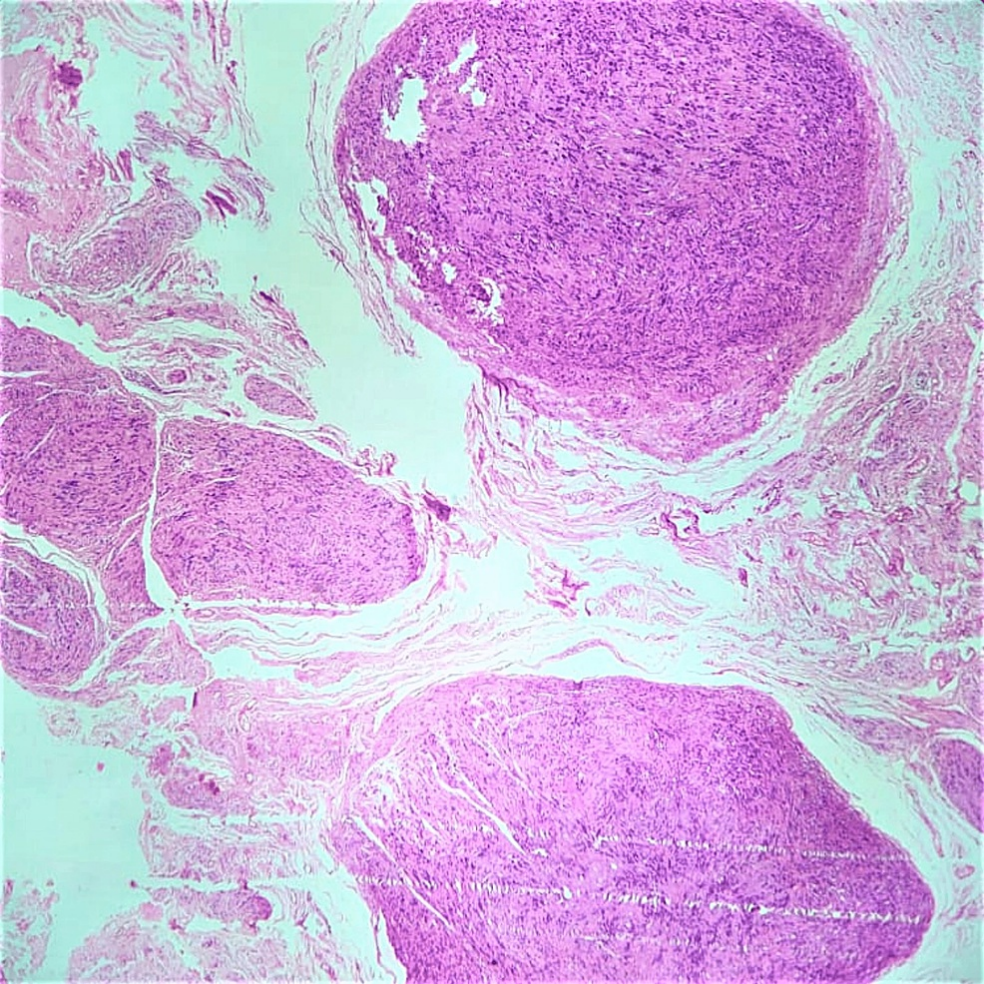
Tumor cells arranged in multiple nodules of varying sizes with a very thin capsule (H&E, 10x) H&E, hematoxylin and eosin

**Figure 4:**
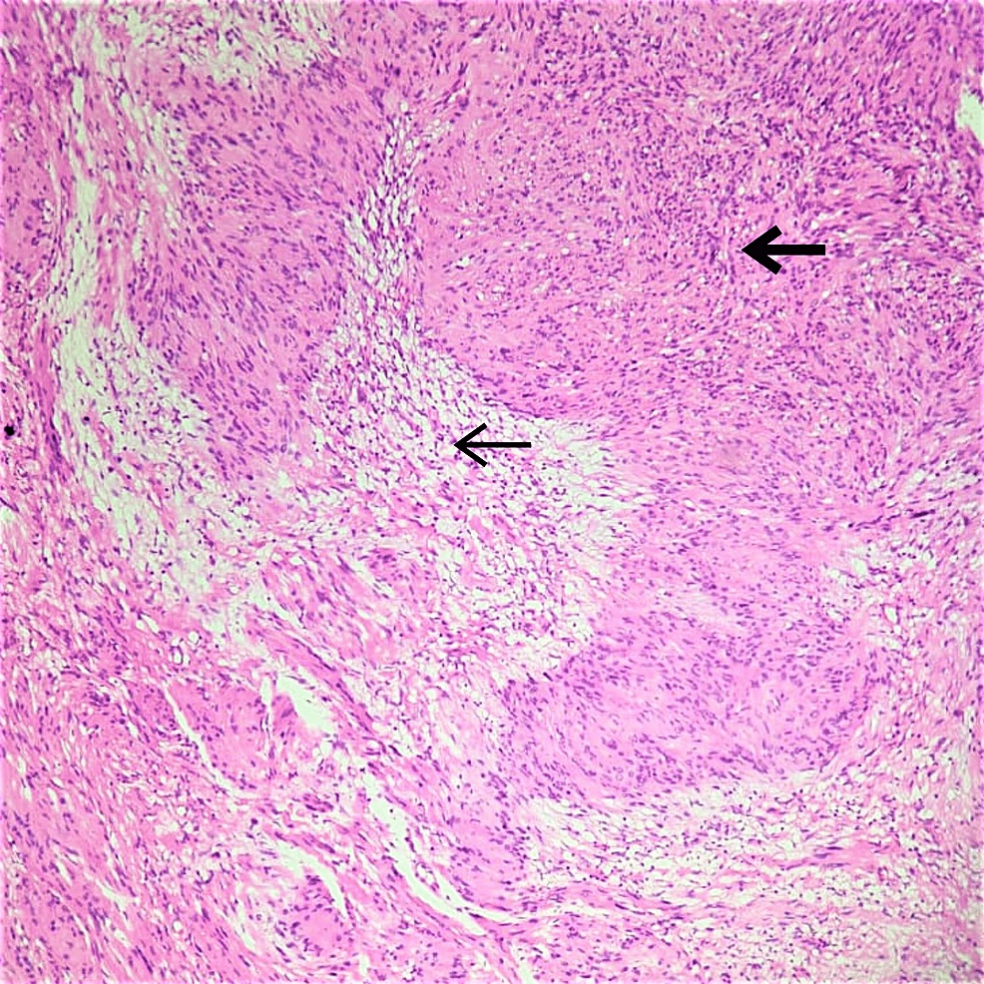
Alternating compact Antoni A (thick arrow) and loose Antoni B (thin Arrow) areas (H&E, 20x) H&E, hematoxylin and eosin

**Figure 5:**
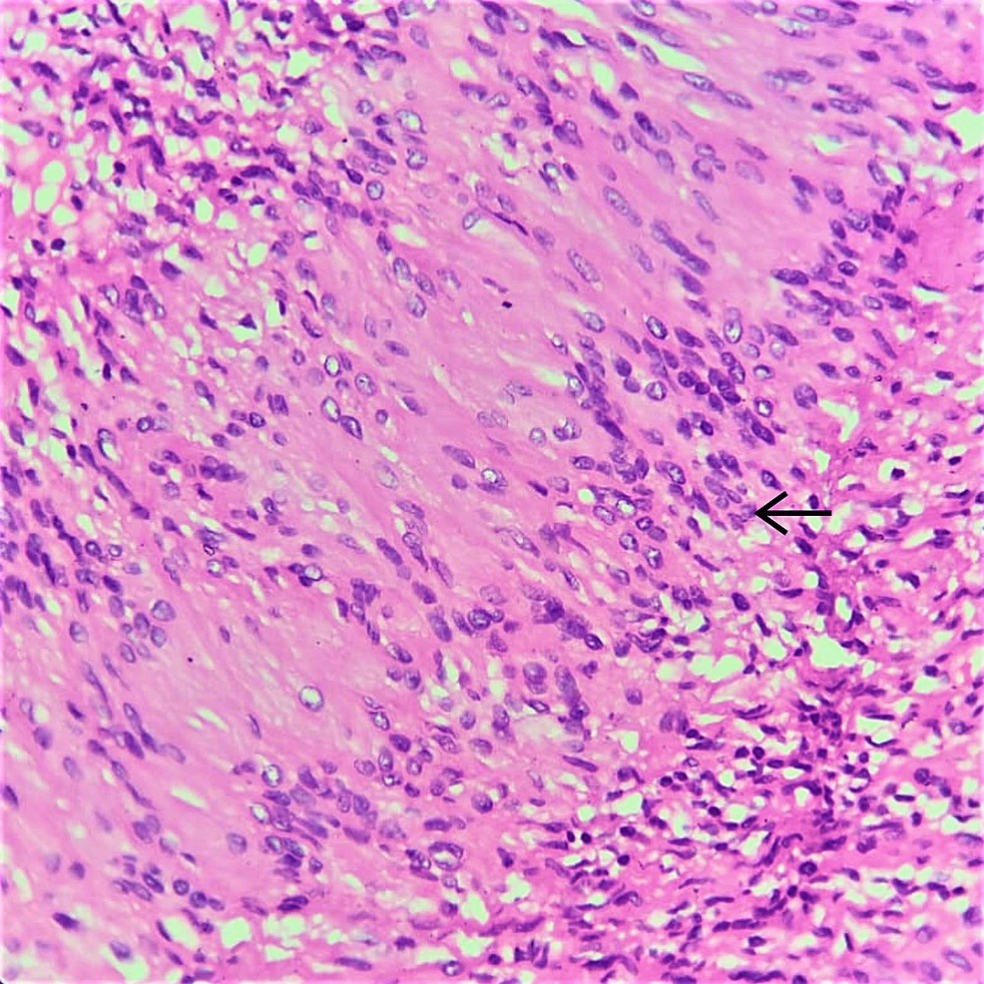
Nuclear palisades or Verocay bodies (arrow; H&E, 40x) H&E, hematoxylin and eosin

A microscopic histomorphological evaluation of formalin-fixed, paraffin-embedded tissue sections stained with hematoxylin and eosin (H&E) was done. Microscopically, sections taken from all three biopsies showed histological features consistent with schwannoma. Sections revealed benign spindle cell neoplasms arranged as multiple nodules of varying sizes with a very thin capsule (Figure 3). The tumor showed hyper- and hypocellular areas (Antoni A and Antoni B types of cells, respectively) along with Verocay bodies (Figures 4, 5). There was no increase in mitosis, necrosis or hyalinized blood vessels, and the final impression was given as features consistent with plexiform schwannoma.

## Discussion

Schwannoma is a well-circumscribed, encapsulated benign neoplasm composed of Schwann cells. They are slow-growing, occasionally painful and typically solitary. Histomorphologically, conventional schwannomas comprise Antoni A areas, containing spindle cells with wavy nuclei, along with parallel rows of nuclei (Verocay bodies). Antoni A areas alternate with hypocellular Antoni B areas, which contain blood vessels in a myxoid matrix [3].

Plexiform schwannoma is a rare variant of schwannoma, comprising only 5% of all schwannoma cases [4]. This variant may show a plexiform or multinodular growth pattern. It can be of either conventional or cellular type. It is associated with NF2 and may also occur in cases of schwannomatosis. Cranial nerves and spinal nerves are generally not affected [1]. Neurofibromatosis Types 1 and 2 are familial autosomal-dominant disorders characterized by tumors of the peripheral and central nervous systems. NF2 is characterized by bilateral vestibular schwannomas, multiple meningiomas, and ependymomas of the cervical spinal cord. Schwannomatosis involves the peripheral nervous system only and may be familial or sporadic. It is characterized by multiple nonvestibular schwannomas, either throughout the body or limited to one region [5].

Plexiform schwannomas harbour somatic loss-of-function mutations in the NF2 tumour-suppressor gene, which is responsible for encoding the merlin protein (schwannomin). The loss of merlin protein expression has been shown to be a consistent finding in schwannomas, irrespective of their allelic makeup or mutations. This implies that the suppression of merlin function is essential for the process of tumorigenesis.

Microscopically, spindle cells in Antoni A patterns are present in the expanded endoneurial space of a neural plexus segment. Individual tumor nodules are surrounded by perineurium. The presence of vascular changes and Antoni type B tissue is variable and cystic changes are rare [2].

Plexiform schwannomas of deep soft tissue may display atypical features such as hypercellularity, pleomorphism, mitotic activity and in rare instances, necrotic and myxoid changes [2]. These atypical components were not present in our case. The neoplastic cells show strong and diffuse positivity for S-100. They also express SOX10 protein, glucuronosyltransferase P (LEU7/CD57), calretinin and may show focal glial fibrillary acidic protein (GFAP) expression [1].

The major differential diagnoses for plexiform schwannomas include malignant peripheral nerve sheath tumors (MPNSTs) and neurofibromas, specifically the plexiform variant. Both MPNSTs and plexiform neurofibromas might show similar findings radiologically, and are therefore mainly differentiated based on their histopathological characteristics. MPNSTs may be multinodular. Unlike plexiform schwannomas, they are infiltrative, highly cellular with elongated cells containing pleomorphic nuclei. They also display a prominent mitotic activity and necrotic changes. It may be problematic to distinguish MPNSTs from the rarer, atypical plexiform schwannoma variants with increased cellularity and mitosis. However, unlike plexiform schwannoma, the tumor cells of MPNSTs stain weakly or negatively with S-100 [1,6].

Plexiform neurofibroma is diagnosed in early childhood and associated with NF1. A total of 2%-5% of cases harbour the risk of malignant transformation [4]. Clinically, they present with grossly enlarged and tortuous nerves. Microscopy shows hypocellularity with myxoid background and no biphasic pattern; occasional nuclear palisading may be noted. Neoplastic cells show scattered S-100 positivity. Perineurial cells are positive for epithelial membrane antigen (EMA) exclusively in the plexiform variant of neurofibroma, whereas they stain negatively in schwannomas [1,6].

The plexiform variant of schwannoma is comparatively more prone to recurrence compared to conventional types. However, the tumor recurrence is limited locally without any metastatic potential, even when associated with rapid growth, hypercellularity or increased mitotic activity [1].

## Conclusions

Plexiform schwannoma is a rare variant of schwannoma. It is crucial but diagnostically challenging to differentiate plexiform schwannomas, which follow a benign course, from MPNSTs, which are highly aggressive and show poor prognosis, and from plexiform neurofibromas that carry a risk of malignant transformation. Microscopic histopathological evaluation and immunohistochemical studies are extremely useful tools for the differentiation of Schwann cell neoplasms.
